# Enhancing Diagnosis of Anterior and Inferior Myocardial Infarctions Using UWB Radar and AI-Driven Feature Fusion Approach

**DOI:** 10.3390/s23187756

**Published:** 2023-09-08

**Authors:** Kainat Zafar, Hafeez Ur Rehman Siddiqui, Abdul Majid, Furqan Rustam, Sultan Alfarhood, Mejdl Safran, Imran Ashraf

**Affiliations:** 1 Institute of Computer Science, Khwaja Fareed University of Engineering and Information Technology, Abu Dhabi Road, Rahim Yar Khan 64200, Punjab, Pakistan; COSC201701011@kfueit.edu.pk (K.Z.); hafeez@kfueit.edu.pk (H.U.R.S.); 2 Cardiology Department, Sheikh Zayed Medical College & Hospital, Rahim Yar Khan 64200, Punjab, Pakistan; drmajidjahangir@hotmail.com; 3 School of Computer Science, University College Dublin, D04 V1W8 Dublin, Ireland; furqan.rustam1@gmail.com; 4 Department of Computer Science, College of Computer and Information Sciences, King Saud University, P.O. Box 51178, Riyadh 11543, Saudi Arabia; mejdl@ksu.edu.sa; 5 Department of Information and Communication Engineering, Yeungnam University, Gyeongsan 38541, Republic of Korea

**Keywords:** myocardial infarction detection, ultra-wideband radar, cardiovascular disease, machine learning

## Abstract

Despite significant improvement in prognosis, myocardial infarction (MI) remains a major cause of morbidity and mortality around the globe. MI is a life-threatening cardiovascular condition that requires prompt diagnosis and appropriate treatment. The primary objective of this research is to identify instances of anterior and inferior myocardial infarction by utilizing data obtained from Ultra-wideband radar technology in a hospital for patients of anterior and inferior MI. The collected data is preprocessed to extract spectral features. A novel feature engineering approach is designed to fuse temporal features and class prediction probability features derived from the spectral feature dataset. Several well-known machine learning models are implemented and fine-tuned to obtain optimal performance in the detection of anterior and inferior MI. The results demonstrate that integration of the fused feature set with machine learning models results in a notable improvement in both the accuracy and precision of MI detection. Notably, random forest (RF) and k-nearest neighbor showed superb performance with an accuracy of 98.8%. For demonstrating the capacity of models to generalize, K-fold cross-validation is carried out, wherein RF exhibits a mean accuracy of 99.1%. Furthermore, the examination of computational complexity indicates a low computational complexity, thereby indicating computational efficiency.

## 1. Introduction

Despite recent developments in the prognosis of diseases, cardiovascular disease (CVD) is a major cause of morbidity and mortality around the globe [1]. The World Health Organization (WHO) reported approximately 17.9 million victims of CVD [2]. CVD is disproportionately prevalent in low- and middle-income countries, where it accounts for approximately 75% of all fatalities [2]. CVD patients experience heart attacks and strokes as the primary causes of mortality, constituting over 80% of all deaths related to CVD. Various notable risk factors, such as an inadequate dietary pattern, insufficient engagement in physical activities, and the consumption of alcohol and tobacco, can lead to an elevated risk of CVD.

Myocardial infarction (MI) is among five manifestations of coronary heart disease (CHD), which include angina pectoris (both stable and unstable), MI, heart failure, and sudden death [3]. Acute MI has been regarded as the most severe manifestation of CHD, with 2.4 million deaths in the United States (US) and 4 million deaths in Europe and Northern Asia [4,5]. Similarly, the study [2] reports around 8 million deaths from MI annually. MI is characterized by the sudden cessation of coronary artery function as a result of thrombus blockage at the site of atherosclerotic disease, leading to the destruction of heart muscle tissue [6]. While chest discomfort and shortness of breath are recognized as clinical indicators of MI [7], it is important to note that these symptoms and physical markers lack sufficient sensitivity and specificity in the accurate detection of MI. The condition frequently arises from a decrease in oxygen supply to the cardiac tissues, leading to the formation of blood clots in the coronary arteries [8]. MI has the potential to affect various regions of the heart, including the anterior, inferior, septal, posterior, lateral, inferior–lateral, septal–anterior, and posterior–lateral segments [8,9]. The obstruction-induced insufficiency of nutrients and oxygen leads to myocardial tissue injury [9].

The monitoring of electrocardiograms (ECGs) and timely detection of abnormalities play a crucial role in reducing mortality rates associated with MI. Single-lead portable ECG monitors are becoming increasingly popular due to their affordable price, effectiveness, and user-friendly interface [7]. ECG data have been utilized for CVD detection in several existing studies [10,11]. Nevertheless, the interpretation of ECG readings can pose challenges due to the intricate and variable dynamics and morphological characteristics of the ECG signal in individuals with MI. The difficulty arises from factors such as the specific area of the heart affected and the extent of myocardial damage. Such factors may lead to delay in the diagnosis or inaccurate diagnosis. The conventional ECG procedure necessitates the placement of electrodes on the patient’s body, which can potentially lead to skin irritation due to the occasional use of conductive gel [12,13]. In addition, electrodes may lead to several skin complications, including skin allergies [14]. Extended usage of the device may result in the occurrence of adhesion and a decline in signal quality, thereby requiring the replacement of electrodes [14].

### 1.1. Significance of Research

**Figure 1:**
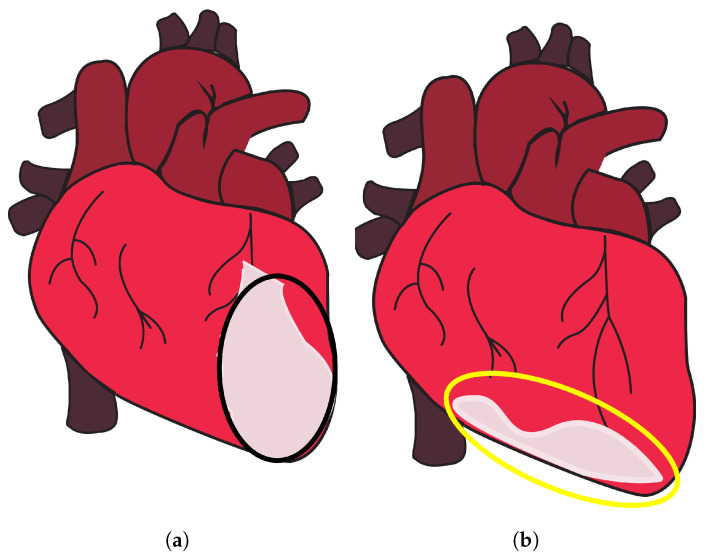
Anterior and inferior MI. (**a**) Part of the heart where anterior MI occurs, encircled in black, and (**b**) part of the heart where inferior MI occurs, encircled in yellow, taken from [22].

This study proposes a non-contact methodology to effectively tackle the challenges and facilitate accurate categorization of anterior and inferior MI. Anterior MI is characterized by myocardial damage that occurs in the front (anterior) region of the heart, specifically affecting the anterior wall of the left ventricle [15], as shown in Figure 1a, encircled in black. The area is supplied with blood by the left anterior descending (LAD) artery, which is a subsidiary of the left coronary artery. The occurrence of an anterior MI can result from the obstruction or occlusion of the LAD [15]. The left ventricle is the largest and strongest chamber of the heart, responsible for pumping oxygenated blood to the body. The contraction of the left ventricle can be significantly impaired as a result of anterior MI [16,17]. The diminished contractility of the left ventricle may result in a decrease in cardiac output, characterized by a reduction in the heart’s capacity to effectively circulate blood [18]. On the other hand, an inferior MI is characterized by infarction of the myocardium that specifically impacts the lower (inferior) region of the heart [19,20], as shown in Figure 1b, encircled in yellow. The predominant source of blood supply to this region is the right coronary artery (RCA), with occasional contribution from the left circumflex artery (LCx) [20]. The occurrence of an inferior MI may lead to detrimental effects on both the right ventricle and the inferior region of the left ventricle [19]. The contraction of the right ventricle is accountable for facilitating the transportation of deoxygenated blood to the lungs, where it undergoes oxygenation.

Studies [15,21] report that patients with anterior MI have a lower ejection fraction than patients with inferior MI. The ejection fraction is a measure of how well the heart pumps blood. A lower ejection fraction means that the heart is not pumping well, which can lead to heart failure [21].

Recent technological advancements offer promising possibilities for the effective detection of anterior and inferior MI. Ultra-wideband (UWB) is a frequently utilized technology in the monitoring of vital signs [14,23,24,25,26,27]. It has several advantageous features over ECG and other technologies. It does not need direct skin contact for patient monitoring, as ECG does. UWB radar employs low power levels and high data rates to produce high-bandwidth signals characterized by extremely brief pulse durations. The device’s capacity to emit a million nano-pulses per second enables it to effectively identify and monitor min movements and vibrations, including respiration and cardiac activity [26,28,29]. Significantly, the IR-UWB radar system is not dependent on visible light or skin complexion, rendering it suitable for deployment in diverse environmental conditions [28,29]. Moreover, the emission power of the device is significantly low, with a limited 41.3 dBm/MHz [30,31,32]. This level of emission poses no harm to human health and remains unaffected by Wi-Fi and cell phone transmissions. The IR-UWB radar possesses a notable advantage in comparison to other existing instruments as a result of its non-intrusive characteristics and its capability to effectively penetrate a wide range of materials and barriers [27,28,29,30,31,32,33].

### 1.2. Major Contributions

Despite the advantages offered by the UWB radar, its efficacy for detecting MI needs to be evaluated. Evidently, the question is: can UWB radar, in conjunction with machine learning (ML) techniques, accurately differentiate between anterior and inferior MI using the contraction magnitude and their specific positions on the heart? The primary objective of this study is to examine the feasibility of utilizing UWB radar in conjunction with ML algorithms for accurately distinguishing between anterior MI and inferior MI. This research endeavors to make a valuable addition to the field of medical diagnostics by improving the diagnostic capabilities and making the following contributions

- This study evaluates the feasibility and efficacy of UWB-based data for inferior and anterior MI detection. Data were collected in a hospital setting from individuals diagnosed with anterior and inferior MI using UWB radar under the supervision of a resident cardiologist. The use of UWB radar in clinical settings provides a practical and real-world perspective to the study.
- The study presents an innovative method for feature fusion, which integrates temporal and class prediction probability features obtained from the spectral feature dataset. The integration of these characteristics seeks to enhance the efficacy of ML models.
- Signal processing techniques were employed to perform preprocessing and enhance the raw UWB data. Afterward, feature extraction techniques were utilized to extract relevant spectral features. For experiments, different ML models were deployed, including random forest (RF), the k-nearest classifier (KNC, logistic regression (LR), Gaussian Naive Bayes (GNB), support vector machine (SVM), and long short-term memory (LSTM).
- Performance of the approach is analyzed, using parameters like accuracy, F1 score, etc. In addition, k-fold cross-validation is also performed to check the robustness of the approach. Performance concerning computational complexity is also employed.

The rest of the article is organized as follows. Section 2 comprises the review of relevant literature. The methodology is explained in Section 3 and experimental results are presented in Section 4. Finally, Section 5 provides the conclusion.

## 2. Literature Review

MI is a prevalent cause of mortality and morbidity around the globe. The early detection of MI is of utmost importance in the effective management and screening of patients. Unfortunately, the initial diagnosis of patients experiencing chest pain often leads to inappropriate admissions, leading to instances where patients without MI receive treatment, whereas those with MI may be overlooked. The utilization of physical examination, precise ECG findings, evaluation of cardiac troponins, and careful consideration of the patient’s medical history are all crucial factors in the timely identification of MI. AI has revolutionized medical fields by enhancing diagnostic accuracy through image analysis and data-driven disease prediction [34,35,36,37,38]. Researchers have developed a variety of techniques to detect distinct types of MI. For example, an 11-layer-deep convolutional neural network (CNN) is presented in [39] for automated MI diagnosis. ECG signals dataset from the Physikalisch-Technische Bundesanstalt (PTB) is used for experiments. The Daubechies wavelet 6 mother wavelet function is used to minimize noise and eliminate baseline wander, and the Pan–Tompkins approach is used to find R-peaks. For noisy ECG signals, the accuracy rate, sensitivity, and specificity are 93.53%, 93.71%, and 92.83%, respectively. The average accuracy, sensitivity, and specificity for noise-free ECG signals are 95.22%, 95.49%, and 94.19%, respectively. As proposed in [40], a variety of cardiovascular disorders, including infarction and arrhythmias, can be identified using limited ECG leads. The timely identification of arrhythmias allows healthcare professionals to swiftly respond, potentially mitigating severe outcomes such as strokes or cardiac arrests. The authors use the well-known PTB dataset, which contains 30-s recordings using 12-lead ECG. The ResNet++ model is used with three leads, II, III, and aVF, to identify inferior and anterior wall MI. The proposed model shows F1 scores of 87% and 89%, exceeding ResNet, which has F1 scores of 84% and 87% for inferior and anterior wall MI, respectively.

The study [41] developed a strategy for detecting inferior MI quickly and accurately. The method analyzes the segmented multi-lead ECG data with the stationary wavelet transform and splits the signal into separate sub-bands. The multi-lead ECG bands are analyzed for parameters such as estimated sample entropy, normalized sub-band energy, log energy entropy, and median slope. SVM and KNN classifiers are used to detect MI patients. Results indicate that KNN produced a receiver operating characteristic curve (ROC) of 99.45%, sensitivity of 98.67%, specificity of 98.72%, positive predictivity of 98.79%, and accuracy of 98.69%. The results are considerably better when SVM is used, with an ROC of 99.94%, sensitivity of 99.35%, specificity of 98.29%, positive predictivity of 98.41%, and accuracy of 98.84% for the class-oriented approach. The subject-oriented technique, on the other hand, produced an average accuracy of 81.71%, sensitivity of 79.01%, specificity of 79.26%, and positive predictivity of 80.25%.

Another study [42] presents an automated technique for detecting Posterior MI (PMI) utilizing a 3-lead vectorcardiogram (VCG). This strategy makes use of electrical conduction properties of heart tissue that vary spatially. A cost-sensitive weighted SVM (WSVM) classifier was devised to solve the issue of class imbalance. The suggested technique was validated using the PTB diagnostic dataset. The WSVM classifier with the radial basis function (RBF) kernel achieved 96.67% test accuracy, 80% sensitivity, and 88.72% geometric mean, respectively. The authors proposed a novel approach in [43] for diagnosis based on the harmonic phase distribution pattern in ECG data. The phase distribution pattern of the Fourier harmonics revealed changes in the shape and timing of the ECG waveform caused by MI. LR and a threshold-based classification approach were used to differentiate between normal and MI participants. The proposed method effectively recognized various types of MI with an average detection accuracy rate of 95.6%, sensitivity of 96.5%, and specificity of 92.7%.

The authors described a unique approach for detecting MI from 12 to lead ECGs [44]. This method made use of a one-of-a-kind hybrid network known as the multiple-feature-branch convolutional bidirectional recurrent neural network (MFB-CBRNN). The study also established an optimization approach called lead random mask (LRM) to address potential overfitting difficulties and increase MI detection accuracy. This strategy lowered the likelihood of overfitting and allowed for the use of implicit ensemble techniques such as dropout. The trials were carried out on the PTB dataset, which included 148 people with MI and 52 healthy people, utilizing class- and subject-based fivefold cross-validation. In class-based tests, the MFB-CBRNN attained an outstanding accuracy rate of 99.90% and 93.08% in subject-based trials. Using single-lead ECG data, ref. [45] reported an automatic and exact approach for MI diagnosis and localization. The solution used a sparse autoencoder (SAE)-based feature extraction network to handle the vanishing gradient problem layer by layer. This enabled the network to find optimal feature expressions for the input heartbeats in the absence of labels. The TreeBagger classifier was then used to diagnose MI by merging the outcomes of many decision trees and improving the diagnostic features. On the PTB dataset, this approach surpassed previous algorithms with an accuracy of 99.90%, sensitivity of 99.98%, and specificity of 99.52%.

The study [46] used 12-lead ECGs to establish two techniques for MI detection and localization. For feature extraction and classification, the first method used discrete wavelet transform (DWT) in conjunction with principal component analysis (PCA) and a shallow neural network (NN). The second method applied an end-to-end deep learning approach to the processed input signals, employing a CNN. In comparison to prior investigations, the models detected MI with an accuracy of over 98% using smaller feature sets. A multi-channel, multi-scale, two-phase deep learning-based technique for MI detection utilizing VCG signals was proposed in [47]. VCG data was decomposed into five components along each channel, resulting in a multi-channel multi-scale tensor input for a CNN. The technique successfully classified MI cases into distinct sub-diagnoses with 99.58% accuracy, 99.87% specificity, and 99.18% sensitivity, respectively. Similarly, ref. [48] also provided an automatic MI detection system based on a CNN model. The CNN model was optimized using a novel loss function known as the concentrated loss. On the PTB dataset, the suggested technique achieved good accuracy, precision, F1 score, and recall of 98.84%, 98.31%, 97.92%, and 97.63%, respectively.

Utilizing ECG data from the PhysioBank database, the study [49] implemented a multi-scale deep learning model with residual networks and attention mechanisms. This model analyzed the 12-lead ECG recordings, calculating and displaying the significance of each lead using the SENet model and Grad-CAM algorithm. By utilizing known MI patterns in specific ECG leads, the model was able to diagnose ten distinct varieties of MI. The outcomes demonstrated extraordinary performance, with average accuracy, sensitivity, and specificity values of 99.98%, 99.94%, and 99.98% for MI detection and 99.79%, 99.88%, and 99.98% for MI localization. The study [50] describes a computerized diagnostic system that detects and classifies five forms of MI from multi-lead ECG signals using a Rough Set classifier. The pathological ECG characteristics associated with MI are extracted, and an information table and knowledgebase are generated. The system identifies critical attributes and generates precise classification rules for MI. It demonstrates robustness via five-fold cross-validation and outperforms existing methods with 99.75% sensitivity and 99.8% accuracy for MI detection and 99.8% accuracy for MI classification. The study [51] examines the significance of accurate ECG in MI diagnosis and ML for automated MI classification. The research utilized Grad-CAM to visualize influential ECG leads and segments in model decisions, with Lead V4 being the most active. Using ECG data from the PTB database, DenseNet and CNN models were developed, achieving high classification accuracy (over 95%). DenseNet outperformed CNN due to its lower computational complexity and higher precision.

The study [52] investigates the detection and localization of MI using ECG signals, a non-invasive and cost-effective diagnostic instrument. Using an RF classifier with 100 trees, it obtains remarkable results: an accuracy of 80.98%, a sensitivity of 80.98%, a specificity of 96.32%, a positive predictive value of 79.72%, and an F-score of 79.53% for MI localization in the interpatient scheme, outperforming existing methods. In the interpatient scheme, it achieves a remarkable 96.54% accuracy, 99.74% sensitivity, 96.09% positive predictive value, and an F-score of 97.88% for MI detection. Even with only six chest leads, the method obtains an interpatient detection accuracy of 96.68%. The study presented in [53] aims to increase the efficacy of MI detection by reconfiguring localization as a multilabel classification problem. ST-deviation, T wave amplitude, and R-S ratios are extracted and implemented in an RF chain classifier with five target classes representing MI locations. The method obtains an impressive overall hamming accuracy of 81.49% in cross-validation tests, with the maximum accuracy for the anterior class at 97.72%.

The above-discussed research works indicate that high-accuracy results can be obtained using ECG data. The conventional implantation of ECG electrodes necessitates the use of conductive gel and may result in skin discomfort and potential health risks. Similarly, in the prevalence of epidemics, a non-contact technology is desired to monitor patients remotely. In order to address these concerns, a novel approach utilizing UWB signals is proposed to distinguish between inferior and anterior wall MI without physical contact. This approach aims to provide a practical solution for remote monitoring in rural settings where conventional ECG placement may pose challenges or be inaccessible.

## 3. Materials and Methods

### 3.1. Proposed Methodology

**Figure 2:**
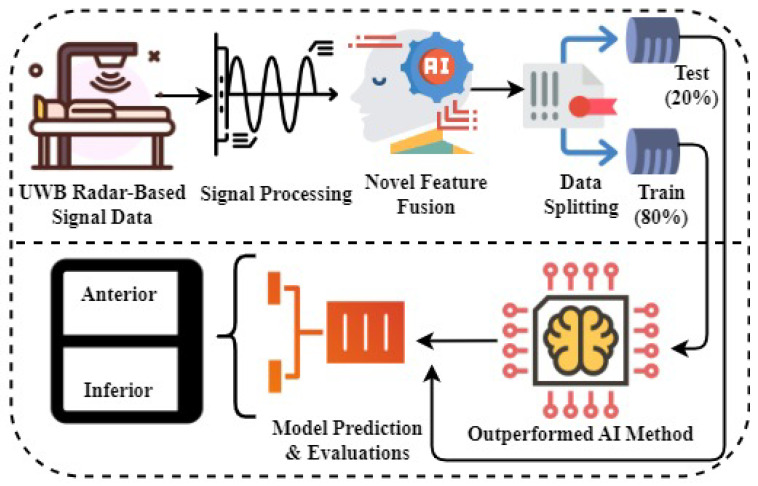
The proposed research methodology and workflow analysis.

This section introduces a novel research methodology for the detection of patients with inferior and anterior wall MI utilizing UWB radar. A UWB radar-based signal dataset relevant to these MI conditions was first gathered in the step-by-step approach, shown in Figure 2. Subsequently, advanced signal processing techniques were employed on the dataset to efficiently eliminate noise and ensure the integrity of the data, thereby enhancing the overall quality of the data for accurate analysis. It was followed by an innovative method for feature fusion, which integrated temporal features and class prediction probabilities. This integration yielded a comprehensive feature set that effectively captured the fundamental characteristics of the signal. The dataset was divided into training and test subsets, with 80% of the data allocated for training. The effectiveness and generalization capabilities of the developed ML models were assessed by validating model performance using the remaining 20% of unseen test data. The ML model that exhibited greater accuracy and efficacy in identifying patients with inferior and anterior wall MI utilizing UWB radar signals was chosen for diagnostic tasks based on extensive performance testing. The utilization of this particular research methodology exhibited the potential of UWB radar in facilitating reliable and precise identification of patients with anterior and inferior MI conditions.

### 3.2. Data Collection

**Figure 3:**
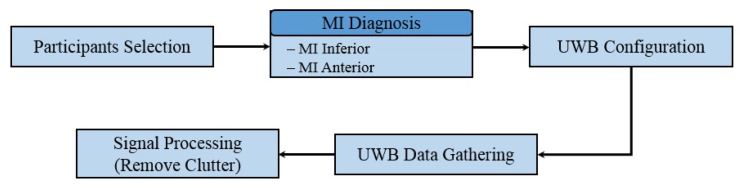
Steps for data gathering using UWB radar.

The data collection process involved the participation of patients diagnosed with inferior wall MI and anterior wall MI at Sheikh Zayed Medical College and Hospital (SZMC and H), Rahim Yar Khan, Pakistan. The data collection was carried out under the supervision of a resident cardiologist. The ethical approval statement was obtained from the ethics committee of the Khwaja Fareed University of Engineering and Information Technology (KFUEIT), in accordance with the criteria outlined in the Helsinki declaration. Consent was obtained from each participant for this study and participants signed a consent form. Figure 3 shows the steps carried out for data gathering.

**Figure 4:**
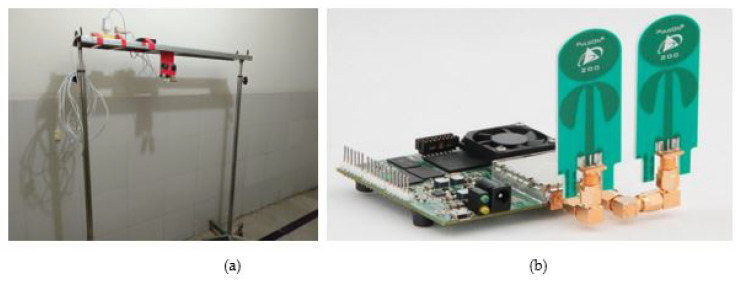
(**a**) Stand designed to mount radar (**b**) PulseON time domain 410 UWB radar.

In order to streamline the process of gathering data, a demonstration was carried out for the cardiologists. Subsequently, the suggestions by the cardiologists were incorporated, and a configuration, as illustrated in Figure 4, was designed. Figure 4a shows the developed setup to properly position the PulseON time domain 410 (P410) UWB, and Figure 4b shows UWB radar used for data collection.

The radar system employed a monostatic arrangement, wherein distinct transmit and receive antennas were positioned in close proximity. The device adhered to the regulations set by the Federal Communications Commission (FCC) for emitting radio waves within the frequency range of 3.1 GHz to 5.3 GHz. The emitted waves were centered at 4.3 GHz and have a bandwidth of 2.2 GHz.

For MI data collection at the hospital, a total of 926 participants were selected in consultation with the cardiologists. The participants included 655 males and 271 females. The age of the participants varied between 40 years and 70 years. Among these individuals, 479 had anterior MI, and 447 had inferior MI, which was diagnosed by the cardiologists. The MI patients were diagnosed by the cardiologists through the interpretation of the electrocardiogram (ECG) recording, which involved recognizing ST-elevations that were indicative of anterior and inferior MIs. Complete details of the dataset are given in Table 1.

**Figure 5:**
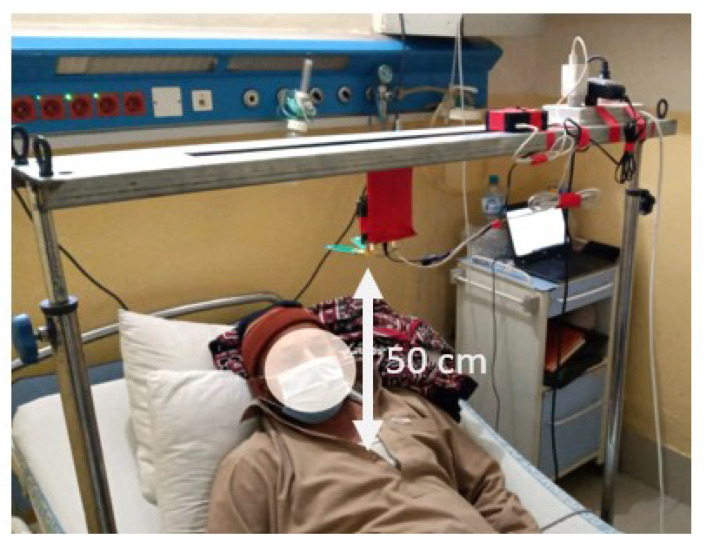
Subject during data collection.

During data collection, the participants were instructed to take a comfortable position on the bed. Subsequently, the radar device was affixed to a frame positioned approximately 50 cm above their chests, as depicted in Figure 5. The radar system was linked to an interface via a Raspberry Pi (RPi) device, which was operated via a virtual network computing (VNC) viewer, facilitating remote control and access to the computing system. Data were collected for a duration of three min for each patient and subsequently stored in distinct folders that were appropriately labeled as “anterior” and “inferior wall myocardial infarction”.

### 3.3. Signal Processing and Feature Extraction

The 3 min radar scan was presented in the form of a matrix, wherein each column corresponded to a vector representing the radar return signal in the fast time domain. Similarly, each row of the dataset corresponded to a vector representing the radar return signal in the slow time domain corresponding to a fast time. The radar scan of three min was divided into segments of the 1 min scan. The radar’s indoor scan range was a distance of 9.5 m, yielding a total of 1440 columns, wherein each column corresponded to a distinct distance from the radar. The measurement of the distance represented by a single column was derived by:

Total scan distance in centimeters = 950 cm
Total columns = 1440
Distance each column covers = 950/1440 = 0.659 cm

The radar scan results in this study were presented in a matrix format, where each column represented a radar return signal vector that spanned 0.659 cm from the radar. Based on the proximity of the subject’s chest to the radar, which was estimated to be approximately 50 cm, a specific column range of 70 to 100 (19.1 cm) was chosen to effectively represent the area surrounding the heart. The range under consideration was presumed to encompass a region where the signals originating from the heart were anticipated to be detectable. It provided vital information for the analysis of MI affecting the anterior wall and inferior wall. The data underwent a two-pulse canceller procedure, as outlined in Equation (1), in order to efficiently minimize the presence of clutter.

(1) $$R_{output}=R_{i}-R_{i-1}$$

**Figure 6:**
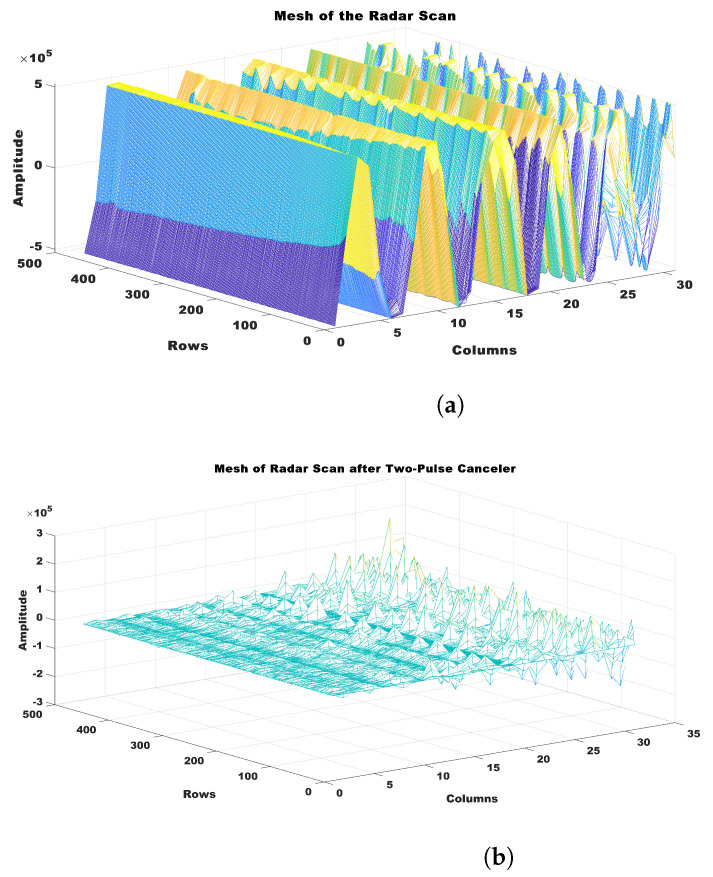
Illustration of pulse canceler. (**a**) Radar scan before pulse canceler, and (**b**) radar scan after application of pulse canceler.

The outcome of this procedure yielded an output signal, denoted as $R_{output}$, which was derived by subtracting the current radar return signal $R_{i}$ from the preceding radar return signal $R_{i-1}$. It helped to eliminate extraneous elements and undesired distortions present in the data, resulting in a refined and more elucidating radar signal that could be used for subsequent analysis and interpretation. Figure 6 displays the radar scan segment of 1 min, both prior to and subsequent to the implementation of the pulse canceler.

**Figure 7:**
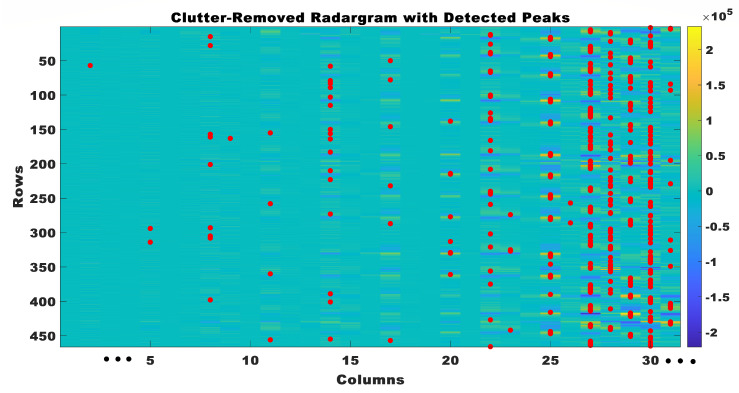
Clutter removed radar grams with detected peaks.

Peak detection was utilized to identify physiological rhythmic windows within the radar data subsequent to the removal of clutter. When the peak detection algorithm identified a peak, it signified the most important point in the radar signal. The peak corresponded to a notable occurrence in the cardiac cycle, such as the contraction or relaxation of the heart. A threshold of 0.4 × 10^4^ was established to differentiate peaks above this threshold in each column, thereby indicating the presence of signals related to the heart. The detected peaks in the clutter-removed radar data are shown in Figure 7.

The physiological rhythmic window was made by combining the values around the detected peaks. The physiological rhythmic window was the temporal window within the radar signal encompassing the rhythmic activity of physiological processes, with its center aligned to the detected peak. The physiological rhythmic window was calculated by collecting data points within a close range of the identified peak in the radar signal, employing a window of size of 10. The computation of the starting and ending indices involved determining a window around the detected peak in the radar data. Its objective was to establish a clear and confined window that remained within the confines of the radar data, thereby preventing any potential errors that may have arisen from the window exceeding the available data. The determination of the initial index entailed subtracting half of the desired window size from the index of the identified peak. This procedure guaranteed that the window effectively captured data points preceding the peak while also ensuring that it did not extend beyond the first column of the radar data.

**Figure 8:**
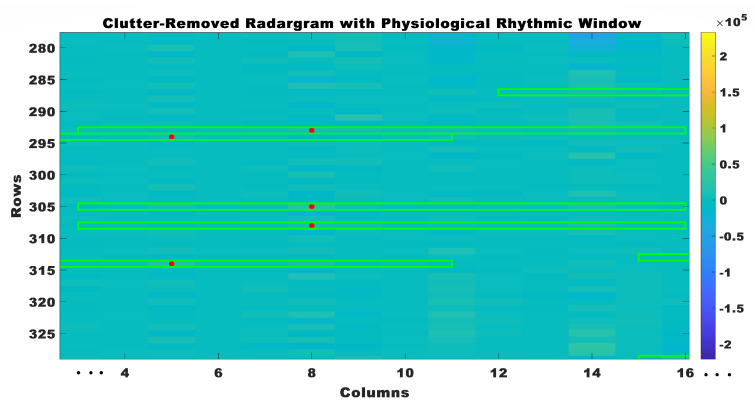
Clutter removed radar grams with physiological rhythmic windows.

In the same manner, the determination of the ending index entailed the addition of half of the intended window size to the index of the identified peak. This methodology ensured that the window effectively captured data points subsequent to the peak while also ensuring that the total number of columns in the radar data was not surpassed. The utilization of the computed initial and final indices effectively established the boundaries of the window encompassing the identified peak. This facilitated the extraction of pertinent data points for subsequent analysis, mitigating any potential challenges associated with data retrieval or indexing errors. The window was positioned at the center of the peak, and data points lying within the window were extracted to construct the physiological rhythmic window, as shown in Figure 8. This procedure was iteratively performed for every identified peak in the radar data, resulting in a collection of distinct physiological rhythmic windows. The construction of physiological rhythmic windows in this manner facilitated a comprehensive analysis of the dynamics of cardiac activity and offered valuable insights into the fundamental physiological processes.

**Figure 9:**
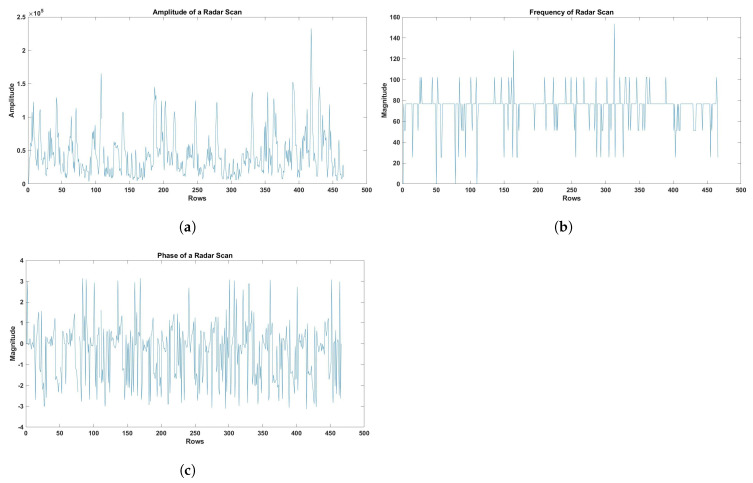
(**a**) Amplitude of the radar scan. (**b**) Frequency of the radar scan. (**c**) The phase of the radar scan.

Afterward, distinct attributes were derived from every individual heart cycle to describe and analyze the cardiac signals. The peak amplitude of the cardiac signal was determined by calculating the maximum value within each heart cycle. This information offered valuable insights into the cardiac response’s intensity throughout the cycle. The frequency component of each cardiac cycle was acquired using Fast Fourier Transform (FFT) analysis. The FFT was utilized to analyze the cardiac signal and discern the distinct frequency components. This enabled the identification of significant frequency components that were associated with the activity of the heart. The phase component of each heart cycle was also acquired through the utilization of FFT analysis. Phase information was a valuable tool for examining the temporal characteristics of the heart’s functioning, as it offered insights into the timing and synchronization of different frequency components within the cardiac signal. Subsequently, the features that were extracted were averaged across all identified physiological rhythmic windows, thereby offering a comprehensive depiction of the cardiac signals from the radar scan. Furthermore, in order to guarantee results that were both meaningful and comparable, the features underwent additional normalization to address any potential discrepancies in signal magnitudes. Figure 9 visualizes the amplitude, frequency, and phase of the radar scan obtained by applying the previously described steps. These features (amplitude, frequency, and phase) were selected in this study, as they were reported to accurately detect and monitor real-time respiratory and cardiac motions [54,55]. For example, amplitude and phase can detect even micromotions of a heart, as reported in [56].

The amplitude, frequency, and phase of each physiological rhythmic window were used to derive a set of key attributes that included energy entropy (EE), spectral entropy (STE), zero-crossing rate (ZCR), root mean square (RMS), centroid frequency (SCF), kurtosis (SK), skewness (SKNS), roll-off (SR), decrease (SD), flatness (SFLT), contrast (SC), flux (SF), spread (SSP), and slope (SSL).The extraction of these features from a signal had great significance, as it enabled the acquisition of useful insights into the distinctive characteristics that were inherent to the signal. The details of these features are given in Table 2. Furthermore, each extracted feature set was assigned a label, denoting the class of the corresponding radar scan, such as anterior MI, represented by 0, and inferior MI, represented by 1. The feature sets, accompanied by their respective labels, were subsequently saved in a CSV file.

### 3.4. Proposed Feature Engineering Approach

**Figure 10:**
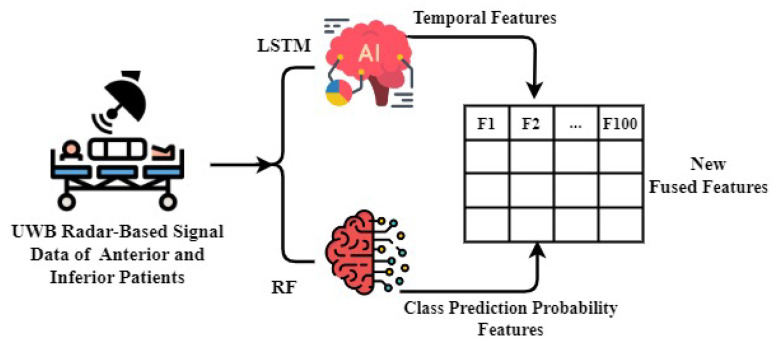
The novel proposed feature fusion mechanism.

A novel feature engineering approach is proposed that combines features to detect patients with anterior and inferior conditions, using signals from a UWB radar. This approach is a key component of the proposed approach. Figure 10 shows the illustration of the working of the proposed feature engineering approach. For feature engineering, the work presented in this manuscript used LSTM and RF models; LSTM was employed for extracting significant temporal features. The authors chose LSTM over Bidirectional Long Short-Term Memory (BiLSTM) due to its simplicity and computational efficiency [57], which are crucial for healthcare applications. LSTM models are widely recognized for their efficacy in capturing sequential dependencies in time-series data, rendering them highly appropriate for analyzing the dynamic characteristics of physiological signals. Concurrently, the preprocessed dataset was fed to the RF model, facilitating the extraction of features related to class prediction probabilities. RF is a robust ensemble learning technique that demonstrates efficacy in managing complicated datasets and offering valuable insights regarding the probability of association in distinct classes.

A novel feature set was generated by combining the features of temporal prediction probability and class prediction probability by LSTM and RF, respectively. The incorporation of this comprehensive set of features enabled us to attain exceptional efficacy in identifying patients with anterior and inferior conditions.

### 3.5. Employed Machine Learning Models

The healthcare and medical industry has undergone significant transformation as a result of the implementation of artificial intelligence (AI) techniques. These techniques have introduced a wide range of applications that have the capacity to revolutionize patient care, diagnostics, and treatment. ML algorithms, including neural networks, have exhibited notable achievements in the field of medical image analysis. These algorithms have proven to be effective in accurately identifying and categorizing diseases based on different types of medical scans such as X-rays, magnetic resonance images (MRIs), and computed tomography (CT) scans. This study investigated the utilization of ML models in the detection of patients with anterior and inferior MI conditions, using a dataset of signals obtained from a UWB radar. In order to attain accurate and effective outcomes, a variety of ML algorithms were utilized, including RF, KNC, LR, GNB, SVM, and LSTM.

The accurate and efficient performance of ML models is heavily influenced by the configuration of hyperparameters. The main focus of this study was to examine the most effective configurations of hyperparameters in order to improve the efficacy of models in identifying anterior and inferior patients. The objective was to enhance the accuracy, sensitivity, and specificity through careful adjustment of these hyperparameters. Table 3 presents the hyperparameters that were chosen through the grid search method, along with their respective values. This table offers valuable information regarding the crucial hyperparameters that had a substantial impact on the performances of the models.

## 4. Results and Discussion

**Figure 11:**
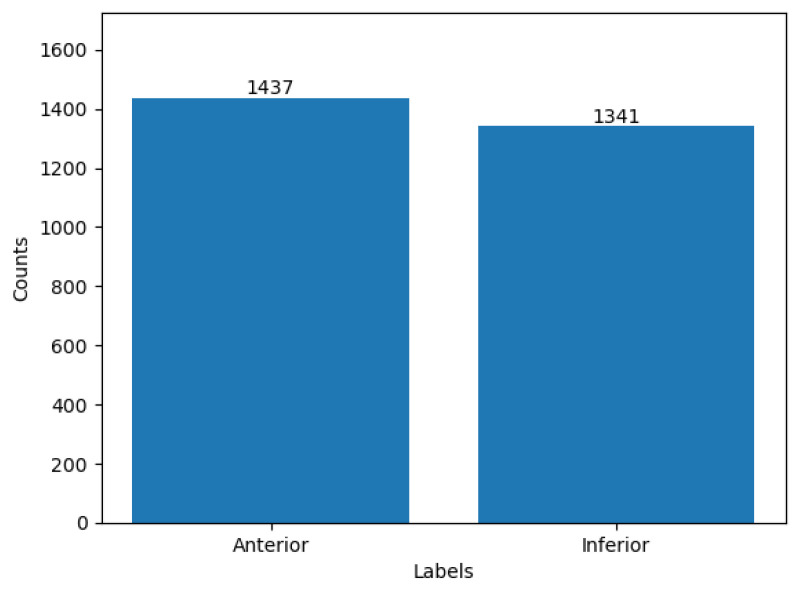
Distribution of class-wise samples.

The utilization of ML methods for identifying anterior and inferior patients yielded encouraging outcomes, as evaluated through essential performance metrics, such as accuracy, precision, recall, and F1 score. The accuracy metric evaluated the general accuracy of the AI-based detection system, whereas the precision and recall metrics offered insights into the system’s ability to correctly classify anterior and inferior patients while minimizing the occurrence of false positives and false negatives, respectively. The F1 score, which considered the balance between precision and recall, provided additional evidence of the efficacy of the model in achieving a balanced and robust detection performance. The analysis of label distribution offered valuable insights into a dataset’s composition and the distribution of patients categorized as anterior and inferior. Ensuring a well-balanced dataset, which included a relatively equal representation of both classes, was crucial in mitigating class bias. This practice contributed to enhanced accuracy and reliability of a model’s outcomes. The label distribution of the dataset is depicted in Figure 11, where the value 0 corresponds to anterior patients, and the value 1 corresponds to inferior patients.

### 4.1. Results Using Original Features

**Figure 12:**
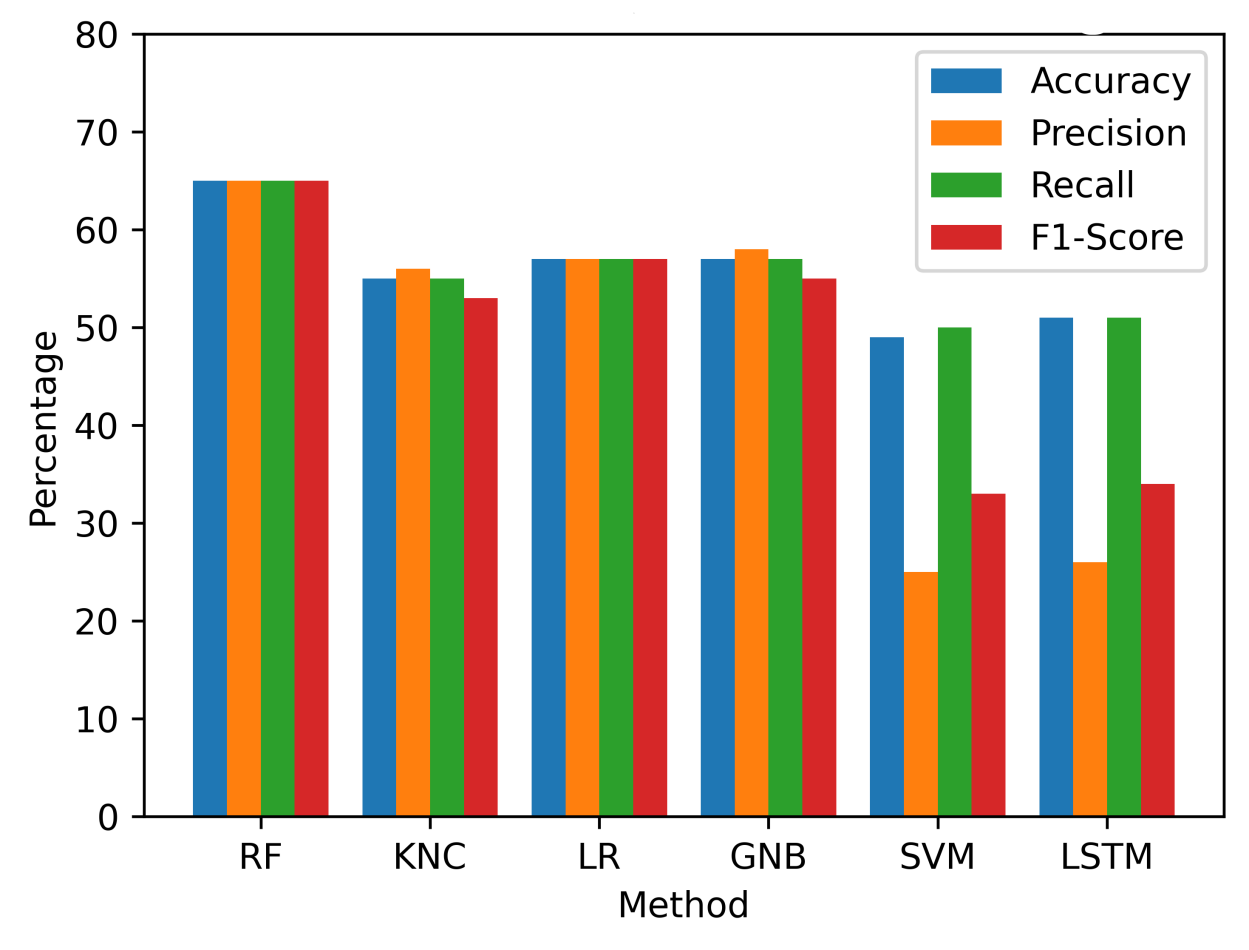
Visualization of performance metrics on the original feature set.

This study employed a preprocessed dataset derived from UWB radar signals to evaluate the efficacy of various models. The analysis demonstrated that the utilization of the employed models with original features yielded suboptimal performance, as shown in Table 4 and visualized in Figure 12. The SVM demonstrated the lowest accuracy of 49%. Similarly, the LSTM, KNC, LR, and GNB models also displayed relatively low performance. Out of the employed models, only the RF model showed a comparatively higher accuracy, reaching 65%. The findings of the initial performance assessment led us to conduct further investigation to gain a more comprehensive understanding of the underlying factors contributing to the below-average results. After conducting a more detailed analysis, it was discovered that the UWB radar-based signal dataset, despite undergoing preprocessing, still exhibited certain complexities and intricacies that presented difficulties for the ML models in accurately capturing and differentiating the intricate patterns that signified anterior and inferior patients. Consequently, a decrease in accuracy and detection rates was observed across all of the implemented models. To tackle these challenges and enhance the efficacy of the models, a comprehensive feature transformation process was implemented.

**Figure 13:**
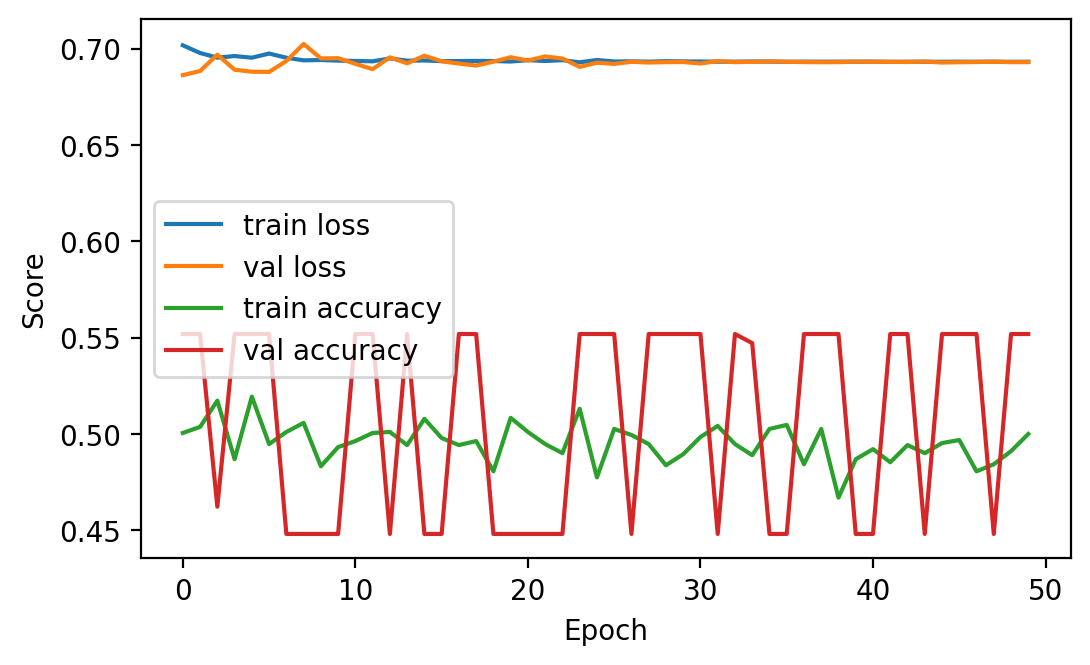
Performance analysis of LSTM.

The performance analysis of the LSTM neural network technique is presented in Figure 13. The analysis indicates that the efficacy of the approach was hindered by the restricted accessibility of data, leading to comparatively low levels of accuracy. During the analysis, it was observed that the training accuracy consistently fluctuated between 50 and 52%, suggesting the presence of difficulties in attaining consistent outcomes. Furthermore, it is worth noting that the loss scores exhibited a persistent elevation throughout the entirety of the 50 epochs, which indicated the inherent challenge in effectively reducing errors during the training phase. The analysis highlighted that the subpar performance of the deep learning approach could be attributed to the insufficient amount of data. Deep learning algorithms typically excel when provided with larger datasets, as this enables them to achieve greater accuracy and generalization.

### 4.2. Results with Proposed Feature Engineering

**Figure 14:**
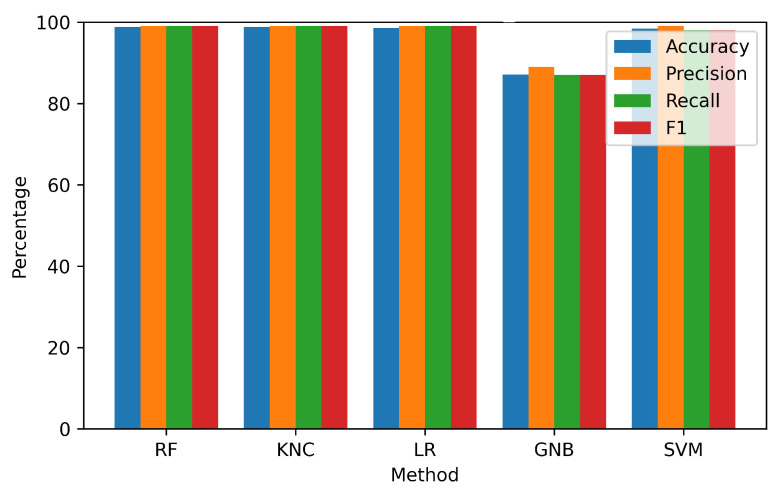
Visualization of performance metrics on the fusion feature set.

This section presents the results obtained through the application of the proposed innovative feature fusion method. The feature sets included both temporal features and class prediction probability features. These feature sets were applied to each of the techniques used in the study. Experimental results are presented in Table 5 and visualized in Figure 14.

The findings demonstrated a significant improvement in the performance of each model when incorporating the features from the proposed approach. Both the RF and KNC approaches demonstrated exceptional accuracies of 98.8% each. However, the GNB approach attained a comparatively lower accuracy of 87.1%. Based on these findings, it can be inferred that the suggested fused feature methodology demonstrated a high level of effectiveness in attaining exceptional performance results for the identification of anterior and inferior MI patients.

### 4.3. K-Fold Cross-Validations Results

**Figure 15:**
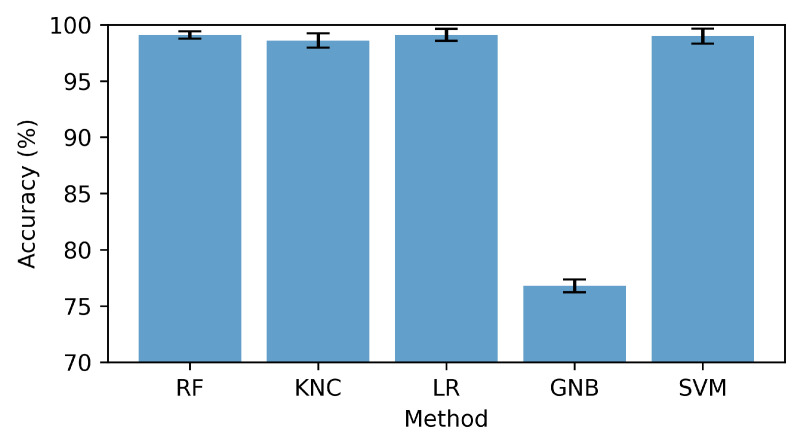
Cross-validation score with standard deviation bar.

The performance evaluation of the utilized methods was carried out in this section by applying k-fold cross-validation. The features were divided into 10 subsets for validation. The findings presented in Table 6 and visualized in Figure 15 demonstrated that the GNB approach yielded a lower k-fold score in comparison, indicating the presence of relatively high standard deviation values. On the other hand, all other applied methods exhibited robust validation scores, thereby highlighting their substantial generalization capabilities. The RF approach stood out among the others due to its exceptional performance. It achieved a remarkable accuracy of 99.1%, accompanied by an impressively low standard deviation score of 0.0033. The findings of this study highlighted the notable efficacy and consistency of the RF approach in precisely identifying anterior and inferior patients.

### 4.4. Computation Complexity

The computational complexity of each model was measured in terms of execution time, as presented in Table 7. The results suggested that the RF model showed the highest execution time of 4.4131 s. It was a tree-based ensemble approach that required higher computation compared to KNC, GBM, and other models. In contrast, the GNB exhibited minimal computational time, suggesting a low level of complexity. Nevertheless, it is important to acknowledge that the RF approach demonstrated superior performance, with a 98.4% accuracy. In general, the analysis demonstrated that the computational complexities of all the models were low and could be used in real-time settings.

### 4.5. Discussion

This research investigated the application of machine learning techniques to UWB radar signal data to identify anterior and inferior patients. Due to the inherent complexities of the dataset, the initial findings with the original features demonstrated difficulties in attaining high accuracy. The size of the dataset posed challenges for deep learning, specifically LSTM. The introduction of a novel feature fusion method that incorporated temporal and prediction probability features, however, marked a significant advancement. This innovative strategy considerably enhanced model performance, with RF and KNC achieving a remarkable 98.8% accuracy. Cross-validation results demonstrated the robustness of these models, particularly RF, whereas computational complexity for real-time applications remained low. The limitations of this work encompassed the relatively modest size of the dataset, thus compromising the generalizability of the findings and, therefore, influencing the performance of the model. The lack of detailed information about the specific features engineered and their clinical relevance also left room for further clarification. Moreover, although the study showcased remarkable precision, the implementation of these models in actual clinical environments presented challenges that went beyond their performances. These challenges included the capacity to understand the models, ensuring compliance with regulations, and taking into account hardware limitations, which were not thoroughly discussed in this study.

## 5. Conclusions

Myocardial infarction is a critical cardiovascular condition that necessitates timely identification and intervention. However, the diagnosis of myocardial infarction can pose significant challenges due to its potential to exhibit a wide range of clinical manifestations and symptoms. Recently, the combination of UWB radar and ML approaches has shown significant potential to improve the diagnosis of various medical conditions. This study aimed to develop a non-contact approach for the detection of anterior and inferior myocardial infarction using UWB radar data. This study collected data in a real-world setting from confirmed patients of anterior and inferior myocardial infarction. The data were refined using signal processing approaches before feature extraction, for which a novel feature engineering approach was designed. The proposed approach integrated temporal and class prediction probability features derived from RF and LSTM models. The experiments involved using several models, like RF, KNC, LR, GNB, SVM, and LSTM, with originals, as well as features extracted using the proposed approach. The experimental results demonstrated that the incorporation of the fused feature set led to a significant enhancement in both the accuracy and precision of models with RF, obtaining the highest accuracy of 98.8%. Further validation was carried out for RF using k-fold cross-validation, and RF showed a 99.1% accuracy. Although the performance of the RF model was better than other models, it showed high computational complexity.

In future work, there are several promising areas to explore in the detection of anterior and inferior myocardial infarction using UWB radar data. An essential factor to consider is the expansion of the dataset by incorporating a broader and more heterogeneous cohort of myocardial infarction patients. It will enhance the generalizability of the proposed approach. The performance and robustness of MI detection can be improved by further refining feature engineering methods and exploring ensemble learning techniques. Moreover, the development of interpretable artificial intelligence models can yield significant insights into the decision-making mechanisms and foster enhanced trust within the medical community.

## Figures and Tables

**Table 1:** Details of the collected dataset.

Item	Details
Participants	Total—926
	Males—655
	Females—271
Age	40 to 70 years
MI type	Anterior—479
	Inferior—447

**Table 2:** Features and their descriptions.

Feature	Explanation
EE	The concept of energy entropy pertains to the evaluation of how energy is distributed among the various frequency components within a given signal. This statement denotes the spectral complexity of the signal and the distribution of energy across various frequency ranges. Greater values of EE indicate a higher level of diversity within the frequency distribution.
STE	Spectral entropy is used to measure the level of uncertainty or randomness in the spectral distribution of a signal. The observed phenomenon is indicative of the wide range of frequencies that are encompassed within the signal. Elevated STE values are indicative of increased spectral complexity and diversity.
ZCR	The zero-crossing rate is a metric that quantifies the frequency at which a signal transitions between positive and negative polarity within a specified time interval. Zero-crossing rate (ZCR) has the potential to facilitate the detection of periodicity or rhythmic patterns within cardiac signals.
RMS	RMS is a metric used to quantify the amplitude of a signal, serving as an indicator of its aggregate energy level. The quantity in question is a mathematical expression that denotes the square root of the average of the squared magnitudes of the signal. A greater RMS value is indicative of a more robust signal.
SCF	SCF represents the spectral distribution’s center of mass. It indicates the signal’s dominant frequency component. Higher SCF values indicate a signal with a higher frequency concentration.
SK	The SK pertains to the quantification of the degree of peakedness, or flatness, exhibited by the spectral distribution. This technique aids in the detection of distinct peaks or anomalous values within the frequency domain. Higher SK values are indicative of a more peaked distribution.
SKNS	The asymmetry of the spectral distribution is denoted by spectral skewness. It represents whether the frequency components are biased toward higher or lower frequencies. Positive SKNS values indicate a frequency deviation toward higher frequencies.
SR	SR represents the frequency below which a specified percentage (e.g., 95%) of the signal’s energy lies. It provides insights into the signal’s frequency content. A higher SR value indicates a higher frequency cutoff point.
SD	Spectral decrease quantifies the decrease in spectral energy with increasing frequency. It can reveal the rate at which the signal’s energy diminishes as frequency increases. A higher SD value indicates a steeper spectral decrease.
SFLT	Spectral flatness measures the flatness of the spectral distribution. It indicates whether the frequency components are evenly distributed or concentrated in certain frequency bands. Higher SFLT values indicate a more uniform distribution.
SC	Spectral contrast measures the difference in amplitude between peaks and valleys in the spectral distribution. It can be useful for detecting specific frequency components or harmonics. Higher SC values signify greater spectral contrast.
SF	Spectral flux quantifies the changes in spectral distribution between consecutive frames. It provides insights into the dynamics of frequency components over time. Higher SF values suggest more significant spectral changes.
SSP	Spectral spread characterizes the spread or dispersion of frequencies in the signal’s spectrum. It can help identify the spread of energy across different frequency components. Higher SSP values indicate wider frequency dispersion.
SSL	Spectral slope represents the rate of change of the spectral distribution. It provides information about the overall trend or slope of the frequency components. A steeper SSL value suggests a more significant change in the spectrum.

**Table 3:** Hyperparameter settings of the AI models.

Method	Hyperparameter Description
RF	max_depth=300, n_estimators=300, random_state=0
KNC	n_neighbors=2, weights=’uniform’, leaf_size=30, p=2
LR	random_state=10, solver=’lbfgs’, max_iter=100, multi_class=’auto’, C=1.0
GNB	priors=None, var_smoothing=1e-09
SVM	penalty=’l2’, loss=’squared_hinge’, tol=0.0001,C=1.0, multi_class=’ovr’, fit_intercept=True, max_iter=100
LSTM	activation=’sigmoid’, optimizer=’adam’, loss=’binary_crossentropy’, epochs=50, metrics=’accuracy’

**Table 4:** The performance results of applied machine learning and deep learning approaches with original signal features data.

Method	Accuracy	Precision	Recall	F1 Score
RF	65%	65%	65%	65%
KNC	55%	56%	55%	53%
LR	57%	57%	57%	57%
GNB	57%	58%	57%	55%
SVM	49%	25%	50%	33%
LSTM	51%	26%	51%	34%

**Table 5:** Performance metrics of models with novel features fusion approach.

Method	Accuracy	Precision	Recall	F1
RF	98.8%	99%	99%	99%
KNC	98.8%	99%	99%	99%
LR	98.6%	99%	99%	99%
GNB	87.1%	89%	87%	87%
SVM	98.4%	99%	98%	98%

**Table 6:** k-fold based cross-validations of models with new fused features.

Method	Accuracy	Standard Deviation (+/−)
RF	99.1%	0.0033
KNC	98.6%	0.0064
LR	99.1%	0.0053
GNB	76.8%	0.0568
SVM	99.0%	0.0066

**Table 7:** Runtime computations of each model (in seconds).

Method	Execution Time (s)
RF	4.4131
KNC	0.0052
LR	0.0213
GNB	0.0078
SVM	0.0189

## Data Availability

Data will be provided on demand.

## Abbreviations

CVD	Cardiovascular Disease
WHO	World Health Organization
CT	Computed Tomography
LAD	Left Anterior Descending
RCA	Right Coronary Artery
LCx	Left Circumflex Artery
AI	Artificial Intelligence
MI	Myocardial Infarction
UWB	Ultrawide band
SZMC and H	Sheikh Zayed Medical College and Hospital
KFUEIT	Khwaja Fareed University of Engineering and Information Technology
UWB	Ultra-Wideband
ML	Machine Learning
ECG	Electrocardiogram
PTB	Physikalisch-Technische Bundesanstalt
CNN	Convolutional Neural Network
VCG	Vectorcardiogram
WSVM	Weighted Support Vector Machine
RBF	Radial Basis Function
SAE	Sparse Autoencoder
FCC	Federal Communications Commission
FFT	Fast Fourier Transform
VNC	Virtual Network Computing
RPi	Raspberry Pi
EE	Energy Entropy
STE	Spectral Entropy
ZCR	Zero-Crossing Rate
RMS	Root Mean Square
SCF	Spectral Centroid Frequency
SK	Spectral Kurtosis
SKNS	Spectral Skewness
SR	Spectral Roll-Off
SD	Spectral Decrease
SFLT	Spectral Flatness
SC	Spectral Contrast
SF	Spectral Flux
SSP	Spectral Spread
SSL	Spectral Slope
RF	Random Forest
KNC	k-Nearest Neighbors Classifier
LR	Logistic Regression
GNB	Gaussian Naive Bayes
SVM	Support Vector Machine
LSTM	Long Short-Term Memory
BiLSTM	Bidirectional Long Short-Term Memory
